# The Potential of Defatted Yellow Mealworm (*Tenebrio molitor*) Meal as an Alternative Protein Source for Juvenile Chinese Mitten Crab (*Eriocheir sinensis*)

**DOI:** 10.1155/2024/8782924

**Published:** 2024-09-30

**Authors:** Wenxiang Yao, Chunyan Zhang, Sai Zhang, Guoan Hua, Sitong Zhao, Huiyang Shuang, Ningyang Sun, Yijun Sun, Lumpan Poolsawat, Jianguo Wang, Quan Wang

**Affiliations:** ^1^ Jiangsu Agri-animal Husbandry Vocational College, Taizhou, China; ^2^ Jiangsu Ocean University, Lianyungang, China; ^3^ Jiangsu Haorun Biological Industry Group Co., Ltd., Taizhou, China; ^4^ Asian Institute of Technology, Bangkok, Thailand

**Keywords:** antioxidant, chinese mitten crab, growth, intestinal health, non-specific immunity, *Tenebrio molitor*

## Abstract

This study was aimed to investigate the effects of replacing dietary fish meal (FM) with defatted yellow mealworm (*Tenebrio molitor*) meal (DYM) on growth performance, intestinal health, serum immune, and antioxidant indexes of juvenile Chinese mitten crab (*Eriocheir sinensis*). Four hundred juvenile Chinese mitten crabs (4.94 ± 0.08 g) were randomly distributed into five groups in quadruplicate (20 crabs per tank), and each group was fed with diets that replaced FM with DYM at 0% (CON, containing 200 g/kg FM), 25% (DYM25), 50% (DYM50), 75% (DYM75), and 100% (DYM100) for 8 weeks, respectively. The results showed that the growth, serum immune and antioxidant indexes, digestive enzyme activities, intestinal histology, and microbiota composition of DYM25, DYM50, and DYM75 groups all reached the similar level as the CON group. While when 100% fishmeal was replaced, significantly decreased the final body weight (FBW), weight gain (WG), serum alkaline phosphatase (AKP), acid phosphatase (ACP), lysozyme (LZM), glutathione peroxidase (GSH-Px), total antioxidant capacity (T-AOC), and total superoxide dismutase (T-SOD) activities, hepatopancreas protease activity, mid-intestine folds height and number (*P* < 0.05), significantly increased the feed conversion ratio (FCR), serum malondialdehyde (MDA) content, and the abundance of intestinal harmful bacteria *Shewanella* (*P* < 0.05). Overall, these results suggest that 75% dietary FM (150 g/kg) can be effectively replaced by DYM without negative impact on the growth performance, intestinal health, serum immune, and antioxidant indexes of juvenile *E. sinensis*.

## 1. Introduction

Chinese mitten crab (*Eriocheir sinensis*) is widely popular among consumers due to its unique flavor and high nutritional value. In 2022, the total production of *E. sinensis* in China reached 815,000 tons [1], making it the highest yield aquatic crustacean species after red swamp crayfish (*Procambarus clarkii*). Under the traditional farming model, the *E. sinensis* primarily fed on chilled fish and oil cake [2]. With the promotion of the green farming model, the breeding proportion based on the whole formula feed is getting higher, and the feed production has increased year by year [3]. Unlike ruminant feed, the diet of *E. sinensis* requires a higher protein content [4], leading to a higher demand for high-quality feed protein sources.

Fish meal (FM) is the highest-quality source of animal protein with well-balanced amino acids and highly digestible, making it the preferred protein source in the diet of *E. sinensis*. However, FM is faced with the problems of resource shortage, unstable quality, and rising price, which has become major difficulties in the application of high-quality formula feed [5]. Consequently, it is imperative to identify protein sources that are not only nutritious but also affordable and consistently available to replace FM. Currently, traditional protein sources like soybean meal [6, 7], rapeseed meal [6, 8], cottonseed meal [9], and fermented soybean meal [3] are still predominantly used as substitutes for FM in the feed of the *E. sinensis*. However, traditional protein sources, whether of plant or animal ingredient, have some substitution problems to some extent, such as poor palatability, amino acid imbalance, lack of active ingredients, and the presence of nutritional antagonists [10].

In recent years, as the insect industry has developed, insect protein has garnered significant interest as a viable alternative to FM. It boasts a comprehensive nutritional profile, lacks anti-nutritional factors, and offers a high feed conversion rate, all of which facilitate easier digestion and absorption by aquatic animals [11, 12]. In the feed industry, yellow mealworm (*Tenebrio molitor*) boasts one of the highest protein nutritive values among insect protein feeds, is known as the “treasure trove of protein feed”, and its industrial high-density farming has been achieved [13]. Additionally, yellow mealworm possesses a well-rounded amino acids composition with the right proportions, a high fat content, and an abundance of polyunsaturated fatty acids, inorganic salts, and vitamins [14]. It is also rich in functional substances like chitin, antimicrobial peptides, and flavonoids [14–16]. The fat content of yellow mealworm meal (YM) can reach 30%, the high fat content is easy to be oxidized, which is not conducive to its preservation, so the manufacturers mostly sell it in the form of defatted yellow mealworm meal (DYM). It has been reported in gilthead sea bream (*Sparus aurata*) [17], rainbow trout (*Oncorhynchus mykiss*) [18, 19], European sea bass (*Dicentrarchus labrax*) [20], Pacific white shrimp (*Litopenaeus vannamei*) [11, 21], common catfish (*Ameiurus melas* Raf.) [22], and narrow-clawed crayfish (*Pontastacus leptodactylus*) [23] that full-fat YM or DYM replaced partial dietary FM without significant effect on growth performance of cultured aquatic animals. However, there have no report on the application of DYM in the diet of *E. sinensis*.

In view of how much FM could be replaced by DYM in practical diet of *E. sinensis*, and what are the effects of replacing FM with DYM on the growth performance, intestinal health, serum immune, and antioxidant indexes of juvenile *E. sinensis*. DYM was used in this study to replace different proportions of FM in a practical diet containing 200 g/kg FM to evaluate the effects on juvenile *E. sinensis*. The results would guide the application of DYM in crab diets.

## 2. Materials and Methods

### 2.1. Experimental Diets and Design

A control diet was designed to contain 200 g/kg FM (Con), and then, 25% (DYM25), 50% (DYM50), 75% (DYM75), and 100% (DYM100) dietary FM were replaced by DYM to form five isoproteic and isolipidic diets, respectively. All ingredients were ground and screened by 60-mesh sieve, and then gradually mixed with fish oil and distilled water (30%) according to the feed formula (as seen in Table 1). The mixture was extruded to form sinking pellets (pelleting temperature of 85 ± 5°C) with a single screw extruder (SLP-22# Dantu district Xin Fenghao construction machinery factory). The pellets were post-cooked in oven at 95°C for 20 min, then air-dried, and stored at room temperature store until use. Amino acids composition of diets, FM, and DYM are shown in Table 2.

FM, DYM, meat and bone meal, peanut meal, cottonseed meal, and brewer's yeast were the main sources of protein in the diet. Fish oil was added to meet the fat needs and flour and corn were added to meet the carbohydrate needs of of *E. sinensis*. The DYM was purchased from Qingdao Sino Crown Biological Engineering Co., Ltd., China, and which crude protein and crude lipid contents were 65.0% and 6.6%, respectively.

### 2.2. Experimental Crab and Feeding Management

*E. sinensis* was obtained from Nanguan river nursery farm (Jiangsu, China), and all crabs were temporarily raised for 1 week in a canvas pool (3.0 m × 1.0 m × 1.0 m, length × width × height) containing disinfected polyvinyl chloride (PVC) pipes as shelter and fed with commercial diet containing 42% crude protein. Then, 400 healthy juvenile crabs (4.94 ± 0.08 g) in the intermolt period with vigorous appendages were randomly assigned to 20 PVC tanks (1.2 m × 1.2 m × 0.75 m) with crab nest as a shelter and circulating water after 24 h of starving. There were five treatments with four replicates (tanks) per treatment and 20 crabs per tank. During the feeding period, the daily feed intake (FI) of about 1%–3% of the body weight was split into two meals fed at 07 : 00 and 17 : 00 for 56 days, and all replicates were feed by hands. The FI was appropriately adjusted based on the water temperature, weather, and feeding behavior of the crabs to ensure the diets could be eaten up in 4 h after feeding. During the cultivation period, the sediment and faeces were removed by siphoning daily, and settled river water was supplemented. The water quality parameters were tested by ProQuatro/1020 (YSI, American), the water temperature, dissolved oxygen, pH, ammonia nitrogen, and nitrite were kept at 23−28°C, ≥5.6 mgL^−1^, 7.0–8.5, ≤0.2 mgL^−1^, and ≤0.05 mgL^−1^, respectively.

### 2.3. Measurement Indicators and Methods

At the end of the feeding experiment, all crabs were deprived of diets for 24 h, and then, the crabs in each tank were counted and weighed with electronic scale (accuracy = 0.01 g). Three crabs were randomly taken from each tank and stored in a −20°C for analysis of crab body proximate composition. After four crabs were anesthetized in ice water, 0.5 mL hemolymph was collected using a 1.0 mL sterile syringe and mixed with the same volume of precooling anticoagulant solution (the formula is sodium citrate 13.2 g/L, citric acid 4.8 g/L, glucose 14.7 g/L). The mixture was immediately centrifuged at 4°C and 12,000 rpm for 20 min to obtain the supernatant and stored in a refrigerator at −80°C. After taking the hemolymph, the hepatopancreas and midintestine were dissected aseptically and frozen immediately in liquid nitrogen, then stored at −80°C for the determination of digestive enzyme activity and intestinal microbial study, respectively. Midintestines of another four crabs from each replicate were dissected and loaded into a 5 mL centrifuge tube filled with 3 mL Bonn's fixative for the analysis of intestinal tissue histology.

#### 2.3.1. Growth Performance

Growth performance indicators including survival, weight gain (WG), feed conversion ratio (FCR), hepatosomatic index (HSI), FI, protein efficiency ratio (PER), and molting interval (MI):

  $$\begin{array}{c} \text{WG }\left(\%\right)=100\times \left[\left(\text{final weight }\left(g\right)-\text{initial weight }\left(g\right)\right)/\text{initial weight }\left(g\right)\right]. \end{array}$$

  $$\begin{array}{c} \text{Survival }\left(\%\right)=100\times \left(\text{final crab number}/\text{initial crab number}\right). \end{array}$$

  $$\begin{array}{c} \text{FCR}=\text{feed intake }\left(g\right)/\left[\text{final weight }\left(g\right)-\text{initial weight }\left(g\right)\right]. \end{array}$$

  $$\begin{array}{c} \text{FI }\left(g/\text{crab}\right)=\text{feed consumption }\left(g\right)/\left[\left(\text{final crab number }+\text{initial crab number}\right)/2\right]. \end{array}$$

  $$\begin{array}{c} \text{PER}=\text{weight gain }\left(g\right)/\text{protein intake }\left(g\right). \end{array}$$

  $$\begin{array}{c} \text{HSI }\left(\%\right)=100\times \text{hepatopancreas weight }\left(g\right)/\text{final body weight }\left(g\right). \end{array}$$

MI is the interval between the date of second molt and the date of first molt (*d*).

#### 2.3.2. Chemical Analyses

Crude lipid, crude protein, ash, and moisture of experiment feed and crabs were measured following the method of AOAC [24]. The moisture content and the ash content were analyzed by drying the samples at 105°C to constant weight and incinerating the samples at 550°C for 6 h, respectively. Crude protein was determined using an Auto Kjeldahl System (2300-Auto-analyzer, Foss Tecator, Sweden), and the crude lipid content was analyzed by the chloroform–methanol method [25].

When determining the amino acid composition of diets, FM and DYM, ~1.0 g of the sample was weighed, 50 mL of 0.1% phenol in 6 mol/L hydrochloric acid was added, ground it into a slurry, and then placed in a 110°C oven for hydrolysis for about 20 h. Removed and cooled, 1 mL of hydrolysate was took, blew with a nitrogen blower until almost dry, 1 mL of 0.1 M hydrochloric acid aqueous solution was added for dissolve, filtered with a filter membrane, and wait for derivative. After derivatization, the sample was filtered with a 0.22 μm needle filter and detect with Agilent 1100 high-performance liquid chromatograph.

#### 2.3.3. Serum Biochemical Indices

Acid phosphatase (ACP), alkaline phosphatase (ALP), lysozyme (LZM), and total antioxidant capacity (T-AOC) were measured using colorimetric methods. The glutamic oxalic transaminase (GOT), glutamic pyruvic transaminase (GPT), total superoxide dismutase (T-SOD), malondialdehyde content (MDA), and glutathione peroxidase (GSH-PX) were determined by visible spectrophotometry, micromethod, xanthine oxidase method, thiobarbituric acid method, and nitrobenzoic acid method, respectively. All indicators were measured according to the instructions of the reagent kit from Nanjing Jiancheng Biotechnology Research Institute, Nanjing, Jiangsu, China.

#### 2.3.4. Digestive Enzyme Activity

Frozen hepatopancreas was mixed with nine times saline (W:V = 1 : 9), then homogenated and centrifuged at 4°C for 15 min (3000 rpm). The supernatant was taken for the determination of digestive enzyme activity. Definition of protease unit: the amount of enzyme that breaks down casein to produce 1 µg of tyrosine per minute at 37°C and pH 7.2 per microgram of tissue protein using 2% casein as the substrate. Definition of lipase unit (µg^−1^ prot): at 37°C, each gram of tissue protein reacts with a substrate for 1 min, and each µmol of substrate consumed is a unit. Definition of amylase unit (µg^−1^ prot): each milligram of protein in the tissue reacts with the substrate at 37°C for 30 min, and hydrolyzing 10 mg of starch is defined as one unit. The protein concentration in the enzyme solution was determined using the Coomassie brilliant blue method, and the test kit was provided by Nanjing Jiancheng Bioengineering Institute (Nanjing, China).

#### 2.3.5. Intestinal Histology

When slicing, the midgut was dehydrated with ethanol, transparentized with xylene, embedded in paraffin, and sliced with a thickness of 5 μm (Leiken RM2235 slicer, Germany), HE staining, sealing to make permanent preservation slices. The morphological characteristics of intestinal folds were observed under a light microscope and photos were taken (Nikon YS100 micrography system). The intestinal epithelial folds height, width, and number were measured using micrometer (the entire midgut cross-section folds number were counted).

#### 2.3.6. Intestinal Microbial Study

The intestinal samples were sent to Shanghai Majorbio Bio-pharm Technology Co., Ltd. for DNA extraction and PCR amplification by Illumina MiSeq Sequencing platform. The V3–V4 variable regions of 16S rRNA gene were amplified by PCR with primers 338F (5′-ACTCCTACGGGAGGCAGCAG-3′) and 806R (5′-GGACTACHVGGGTWTCTAAT-3′). The microbiota community composition and community abundance at phylum and genus levels were analyzed on the free online platform of Majorbio Cloud Platform (https://www.majorbio.com).

### 2.4. Statistical Analysis

The experimental data were presented as mean ± standard deviation. All data were analyzed using SPSS 22.0 statistical software. A one-way analysis of variance (ANOVA) and Turkey's multiple range tests were used to determine the statistical significance among groups. Statistical significance was determined at *P* < 0.05. In addition, a follow-up trend analysis was performed using orthogonal polynomial contrasts to determine whether the significant effect was linear and/or quadratic.

## 3. Results

### 3.1. Growth Performance

As shown in Table 3, significant linear and quadratic effects of FM replaced by DYM were observed in FCR (*P* < 0.05), and significant linear effects were observed in final body weight (FBW), WG, and PER (*P* < 0.05). When the replacement amount of FM reached 100%, the FBW, WG, FCR, and PER were significantly decreased, and FCR were significantly increased compared to the CON group (*P* < 0.05). Compared with CON, no significant difference was observed in SR, HSI, and MI (*P* > 0.05).

### 3.2. Whole-Body Proximate Composition

**Figure 1:**
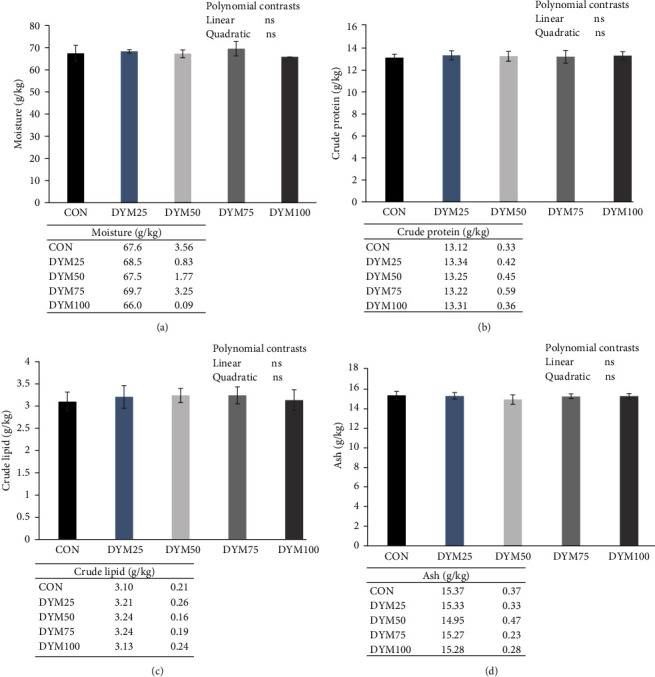
Effects of different substitution levels of FM with DYM on the proximate composition in whole body of *E. sinensis*. (a) Moisture, (b) crude protein, (c) crude lipid, and (d) ash content in whole body of *E. sinensis*. The data are expressed as mean ± standard deviation (*n* = 4). DYM, defatted yellow mealworm meal; FM, fish meal; ns, no significant.

In the whole-body proximate composition, no significant difference was observed among all the treatments (*P* > 0.05, Figure 1).

### 3.3. Digestive Enzymes Activities in Hepatopancreas

**Figure 2:**
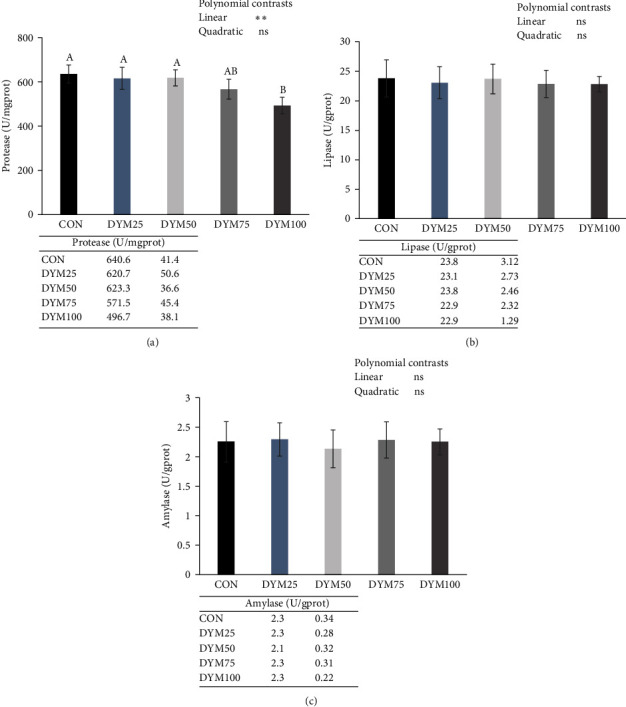
Effects of different substitution levels of FM with DYM on digestive enzymes activities in hepatopancreas of *E. sinensis*. (a) Hepatopancreas protease, (b) lipase, and (c) amylase of *E. sinensis*. The data are expressed as mean ± standard deviation (*n* = 4).  ^*∗∗*^*P* < 0.01. FM, fish meal; DYM, defatted yellow mealworm meal; ns, no significant.

The orthogonal polynomial contrasts showed that the level of replacing FM with DYM linearly decreased the hepatopancreas protease activity, which of DYM100 group was significantly lower than the CON group (*P* < 0.05, Figure 2), but without significant difference on the hepatopancreas lipase and amylase activity (*P* > 0.05, Figure 2).

### 3.4. Serum Biochemical Indices

**Figure 3:**
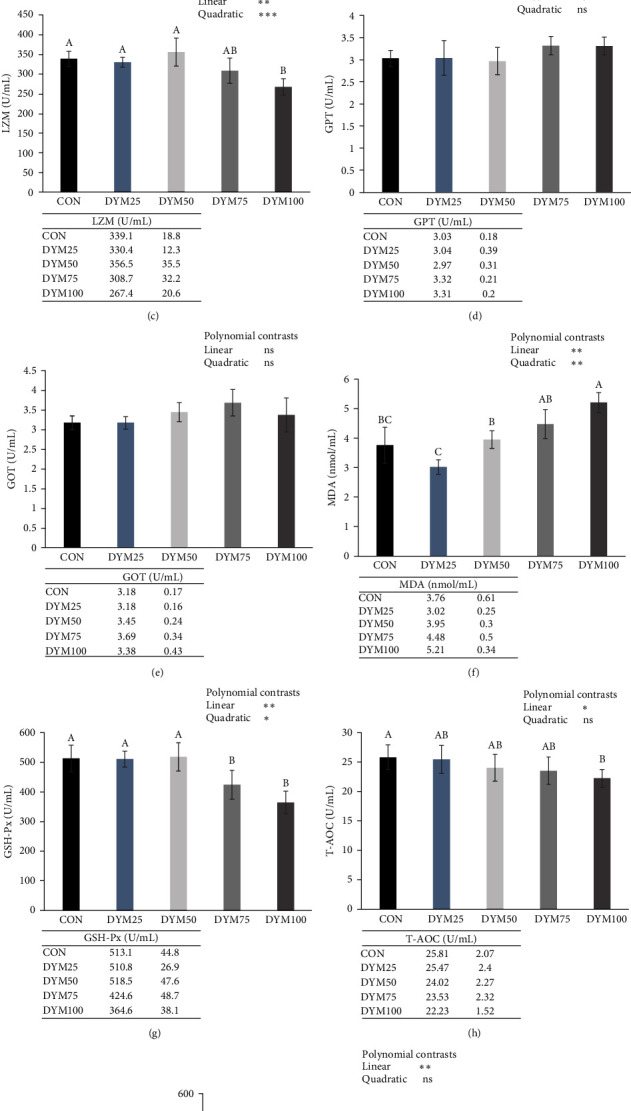
Effects of different substitution levels of FM with DYM on the serum biochemical indices of *E. sinensis*. The data are expressed as mean ± standard deviation (*n* = 4).  ^*∗*^*P* < 0.05;  ^*∗∗*^*P* < 0.01;  ^*∗∗∗*^*P* < 0.001. ACP, acid phosphatase; AKP, alkaline phosphatase; DYM, defatted yellow mealworm meal; FM, fish meal; GOT, glutamic oxaloacetic transaminase; GPT, glutamic-pyruvic transaminase; GSH-Px, glutathione peroxidase; LZM, lysozyme; MDA, malondialdehyde; ns, no significant; T-AOC, total antioxidant capacity; T-SOD, total superoxide dismutase. (a) Serum ACP, (b) AKP, (c) LZM, (d) GPT, (e) GOT, (f) Serum MDA content of E. sinensis, (g) GSH-Px, (h) T-AOC, and (i) T-SOD activities of *E. sinensis*.

As shown in Figure 3, significant linear and quadratic effects of FM replaced by DYM were observed in serum LZM, GSH-Px activities, and MDA content (*P* < 0.05). Significant linear effects of FM replaced by DYM were observed in serum alkaline phosphatase (AKP), T-AOC, and T-SOD activities, significant quadratic effect were observed in serum ACP activity (*P* < 0.05). For immune enzyme activity, ACP, and LZM activities showed a trend of first increasing and then decreasing. Regarding these two indicators, the DYM50 group had the highest activity, while the DYM100 group was significantly lower than the DYM50 group (*P* < 0.05). In terms of antioxidant activity, the T-AOC, T-SOD, and GSH-Px activities of the DYLM100 group were significantly lower than the CON group, and the MDA content was significantly higher than that of the CON, DYM25, and DYM50 groups (*P* < 0.05). In GOT and GPT, no significant difference was detected among all the groups (*P* > 0.05).

### 3.5. Intestinal Histology

**Figure 4:**
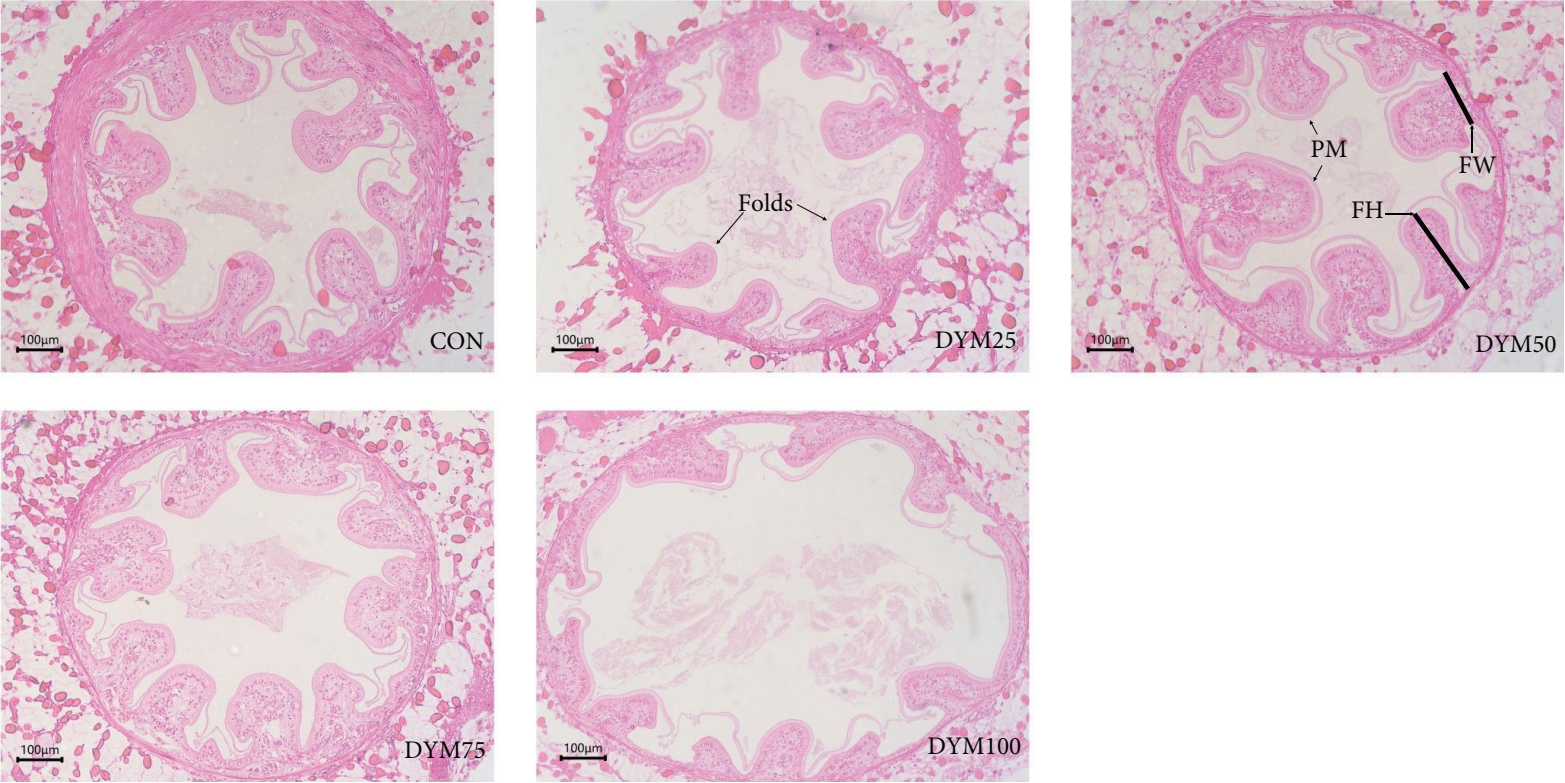
Effects of different substitution levels of FM with DYM on intestinal tissue structure of *E. sinensis*. DYM, defatted yellow mealworm meal; FM, fish meal.

It was showed by the orthogonal polynomial contrasts that the increasing level of DYM led to significant linearly and quadratically decrease of midintestinal folds height, and linearly decrease of midintestine folds number (*P* < 0.05). There was no significant difference in midintestinal folds width and peritrophic membrane thickness (*P* > 0.05). Compared with the CON group, the DYM100 resulted in a significantly lower midintestinal folds number and height (*P* < 0.05), while no significant difference was detected among the other four groups (*P* > 0.05) (Table 4, Figure 4).

### 3.6. Microbial Study in Intestine Using 16 rRNA Sequencing

**Figure 5:**
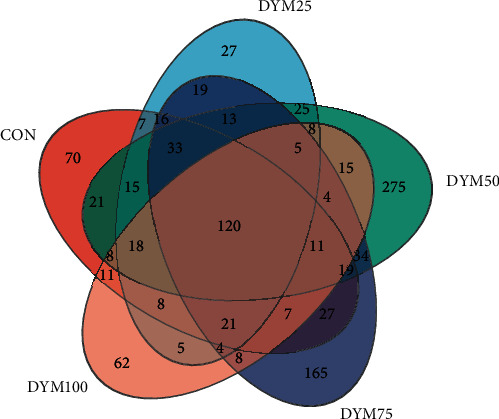
Venn analysis of the OTU numbers of gut microbiota in *E. sinensis* feeding with different diets.

A total of 1,220,830 valid sequences were obtained with sequence lengths ranging from 200 to 512 bp, and the average sequence length was 422 bp. After cluster analysis of the obtained sequences, five groups obtained 412, 344, 624, 506, and 327 representative bacterial communities OTUs, respectively. The OTUs shared by the five treatment groups are 120, while the unique OTUs are 70, 27, 275, 165, and 62, respectively (Figure 5).

**Figure 6:**
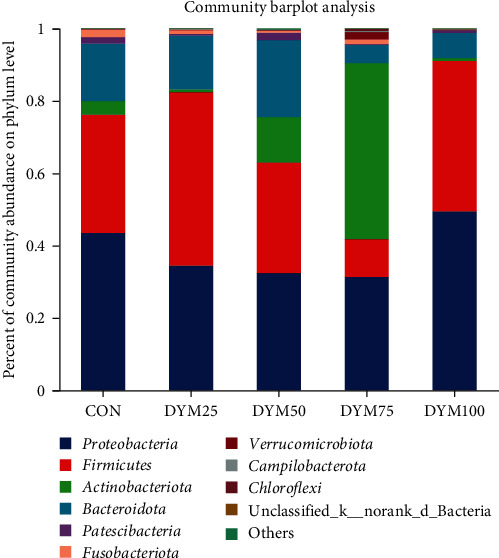
Abundance at the phylum level of gut microbiota in *E. sinensis* feeding with different diets.

**Figure 7:**
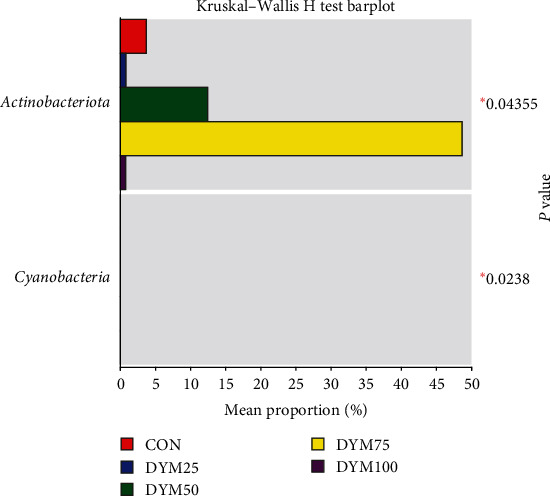
Comparison of difference at the phylum level of gut microbiota in *E. sinensis* feeding with different diets.

At the phylum level, *Proteobacteria*, *Firmicutes*, *Actinobacteriota*, and *Bacteroidota* are the most abundant bacteria in the intestinal microflora of *E. sinensis*, accounting for 95.80%, 98.05%, 96.80%, 95.50%, and 98.81%, respectively (Figure 6). As shown in Figure 7, there were significant differences in the abundance of *Actinobacteria* and *Cyanobacteria* in the microbial composition of each treatment group (*P* < 0.05).

**Figure 8:**
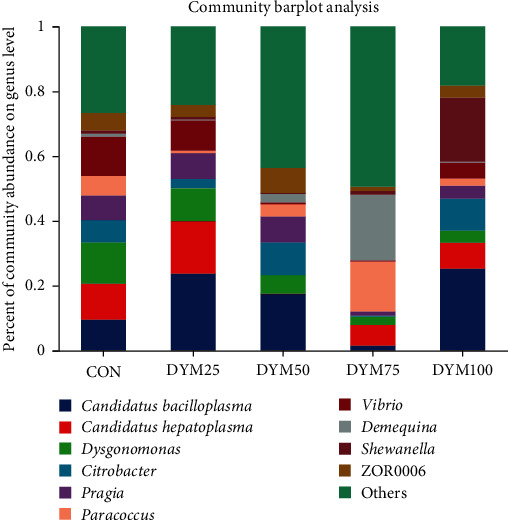
Abundance at the genus level of gut microbiota in *E. sinensis* feeding with different diets.

**Figure 9:**
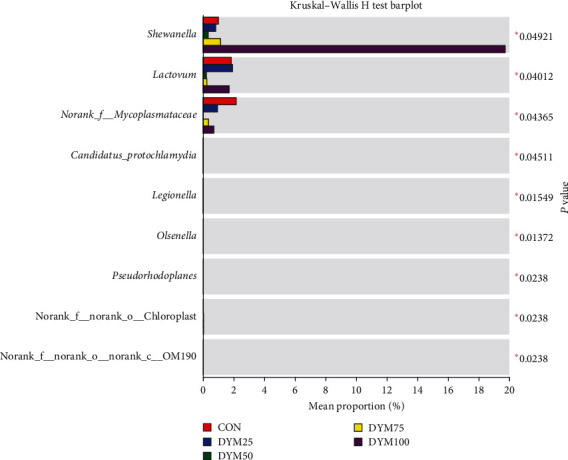
Comparison of difference at the genus level of gut microbiota in *E. sinensis* feeding with different diets.

At the genus level, the composition of dominant gut microbiota varies among different groups. *Candidatus Bacilloplasma* was the most abundant intestinal microflora among all the groups (except for DYM75), followed by *Dysgonomonas* (CON and DYM25), *Citrobacter* (DYM50), and *Shewanella* (DYM100). The dominant intestinal microflora of the DYM75 group were *Demequina* and *Paracoccus* (Figure 8). As the amount of FM replaced by DYM reached 100%, the abundance of *Shewanella* in crab intestinal significantly higher than other groups (*P* < 0.05). In addition, there were also significant differences in the abundance of *Lactovum*, norank_f_*Mycoplasmataceae*, unclassified_f_*Intrasporangiaceae* among all the groups (*P* < 0.05) (Figure 9).

## 4. Discussion

As a high-quality insect protein, YM boasts high protein and fat content. It serves as a promising alternative to FM and has been successfully cultivated in high-density and intensive facilities. In recent years, the feasibility of substituting FM with YM in aquafeeds has garnered significant interest. Numerous studies have established that substituting FM with full-fat YM or DYM notably enhanced the growth performance of aquatic animals [11, 13, 23, 26, 27]. Motte et al. [11] reported that the replacement of 25%, 50%, and 75% dietary FM (the basic feed FM content is 250 g/kg) by DYM significantly improved the growth performance of Pacific white shrimp (with DYM additions of 52, 103, and 154 g/kg, respectively). Similar results also shown in the research of Rema et al. [28] on rainbow trout that replacing FM with 50, 75, 150, and 250 g/kg DYM significantly increased the FBW, SGR, and decreased the FCR (*P* < 0.05). However, in the present study, substituting 25%, 50%, and 75% dietary FM with DYM did not significantly enhance the growth performance of the *E. sinensis*. Furthermore, when the replacement ratio reached 100%, the FBW of the crab was significantly decreased, and the FCR was significantly increased, which is different from the previous reports. Motte et al. [11] and Rema et al. [28] substituted FM with DYM and supplemented exogenous essential amino acids. In contrast, our experiment did not include such supplementation. Consequently, the lack of growth-promoting effect in this experiment might be attributed to the unbalanced amino acid composition, resulting in an imbalanced amino acid profile, which in turn directly impacts the *E. sinensis*'s absorption and utilization of feed amino acids. Panini et al. [21] also indicated that methionine is the first limiting amino acid of full-fat YM, and Khosravi et al. [29] found that substituting FM with full-fat YM and supplementing with methionine improved the growth rate and protein utilization of aquatic animals to a certain extent, which is consistent with our viewpoint.

Furthermore, some scholars found that the high chitin content and its derivatives in YM is a limiting factor in the substituting of FM [30]. Chitin is an important component of the carapace, gut lining, and peritrophic membrane in crustaceans [31, 32]. As an important nutrient, chitin must be degraded by chitinase and utilized to provide nutrients for the body [33]. However, chitinase have only been identified in a few fish species, such as Nile tilapia (*Oreochromis niloticus*) [34] and sea bream [35]. The presence of chitinase in most economic fish species has not been reported, so the high contents of chitin typically reduced the nutrient utilization and growth performance of cultured fish species [33, 36]. Therefore, the use of YM in the diet of fish is constrained by its high chitin content, whereas crustaceans with their habits of cannibalism or molting, are not as constrained as they can secrete chitinase. Nevertheless, the chitin content in YM can reach up to 13.72% [37]. As the proportion of YM in the feed increases, so does the chitin content in the digestive system. The secreted chitinase is insufficient to break down so much chitin, and excessive chitin combines with digestive enzymes, such as leucine aminopeptidase (a brush border enzyme breaking down peptides into amino acids) hindering the digestion and absorption of other nutrients [38]. The present study also found that substituting 25%–75% of the FM with DYM shows no significant effect on the *E. sinensis*'s intestinal tissue structure, while when the substitution reaches 100% (with DYM addition of 216.8 g/kg), the intestinal structure was damaged, manifested by a reduction in both the number and height of the intestinal folds in crabs, thereby reduced the growth performance. Similar results also shown in the research of Hu et al. [39] on sea bass (*Lateolabrax japonicas*) and Li et al. [40] on Jian carp (*Cyprinus carpio* var. Jian). Chitin was reported to induce intestinal inflammation [41]. In summary, we believe that the declining growth performance of the DYM100 group not only related to the imbalanced feed amino acids, but also to the high chitin content.

Digestive enzymes are crucial for nutrient digestion, with their activities directly indicating the digestive capabilities, nutritional status, and growth performance of aquatic animals [42]. Feed is a key factor affecting the activity of digestive enzymes in crustaceans. Sánchez-Muros et al. [43], Melenchón et al. [44], and Zheng [45] discovered that incorporating full-fat YM or DYM into the feed enhanced the intestinal protease activity in tilapia, rainbow trout, and Pacific white shrimp, respectively. Nevertheless, Hoffmann et al. [46] discovered that the supplementation of full-fat YM in the diet of sea trout had no significant effect on the gut digestive enzymes, similar findings were reported in Pacific white shrimp [47]. On the contrary, in this experiment, replacing FM with DYM at levels from 0% to 75% did not significantly affect the activity of digestive enzymes in the hepatopancreas of *E. sinensis*. While the replacement amount of FM reached 100%, the protease activity was significantly decreased. FM as the most superior protein source in aquatic feeds, the high replacement level of DYM led to a reduction in the digestive capacity of *E. sinensis* in this experiment, potentially attributed to lower content or unsuitable ratio of essential amino acids in DYM. Furthermore, other components within DYM might also influence the activity of digestive enzymes in the hepatopancreas, necessitating further detailed research.

The intestine is the longest segment of the digestive tract in aquatic animals and is a critical site for nutrient absorption. Intestinal microbiota is integral to intestinal health, maintaining the integrity of intestinal epithelial cells, and preventing the invasion of pathogenic microorganisms. Moreover, it regulates the intestinal immune system by modulating the secretion of antibodies. Intestinal microbiota plays an important role which is involved in the nutritional metabolism [48] and influences the immune development [49] of host, and its composition is largely influenced by environmental and dietary factors [50–52]. Previous studies have demonstrated that the predominant microbial communities in the gut microbiota of *E. sinensiss* consist of *Firmicutes*, *Bacteroidetes*, *Proteobacteria*, and *Tenericutes* [53–55], with *Tenericutes* being the most abundant. However, in this experiment, the *Tenericutes* were not detected in the gut microbiota of any group of crabs, and the composition of the dominant microbial community is consistent with the research results of Yang et al. [56] and Jiang et al. [5]. In this experiment, the predominant gut microbiota in *E. sinensiss* also included *Actinobacteria*, with significant intergroup differences were observed. *Actinobacteria* are aerobic, gram-positive bacteria that include beneficial species like *bifidobacteria*, crucial for intestinal health, and the dominant genus *Streptomyces*, which degrade polymers like chitin and fiber [57]. Additionally, they harbour various pathogenic bacteria, elevating the risk of intestinal illnesses in aquatic animals [58]. By comparison, what we are more concerned about is the beneficial or harmful gut bacteria at the genus level within animal intestines. Tran et al. [59] found that replacing 75% of the FM in European sea bass feed with DYM led to a reduction in the abundance of *Lactobacillus* which is the beneficial bacteria of intestine. In addition, Zheng et al. [60] discovered that when the proportion of DYM replacing FM in feed reached 45%, the abundance of pathogenic bacteria *Vibrio*, *Spongiimonas*, and *Tenacibaculum* in the intestine of Pacific white shrimp increased. Similarly, this experiment noted a significant rise in the abundance of *Shewanella* in the gut of *E. sinensis* when the substitution of DYM for FM reached 100%. *Shewanella*, a member of the *Vibrionaceae* family, is a facultative anaerobic gram-negative bacterium that can produce H_2_S. It serves as a major conditional pathogen in aquatic animals and proliferates rapidly when the gut microbiota is disrupted, leading to disease outbreaks [61, 62]. Thus, substituting a small amount of FM with DYM in feed does not affect the intestinal health of *E. sinensiss*, when the substitution amount reaches 100%, imbalance intestinal microbiota was caused.

Crustaceans rely solely on their innate immune system to fend off pathogen invasion due to the lack of adaptive immunity [9]. LZM, AKP, and ACP are humoral immune factors and critical immune indicators for the nonspecific immune ability of crustaceans. LZM, produced by neutrophils and macrophages, specifically hydrolyzes *β*−1,4 glycosidic bonds in peptidoglycan [63], which has been demonstrated to have broad-spectrum antibacterial activity against both gram-negative and gram-positive bacteria [64]. ACP is a marker of lysosomal digestion of invading organisms [65], while AKP is a phosphate monoesterase that detoxifies, digests, and absorbs multiple nutrients during normal life and phagocytosis [66]. Previous studies on largemouth bass (*Micropterus salmoides*) [67], giant freshwater prawn (*Macrobrachium rosenbergii*) [13], mandarin fish (*Siniperca scherzeri*) [68] and yellow catfish (*Pelteobagrus fulvidraco*) [69] have shown that the activities of serum LZM, ACP, and AKP increased with the increasing of dietary full-fat YM or DYM content. This may be attributed to the abundance of the chitin in YM, which possesses the capability to stimulate innate immune cells and bolster the organism's immune response by inducing the production of cytokines via diverse cell surface receptors [36]. However, in the present study, when DYM was used as a substitute for FM at 25%, 50%, and 75%, there was no significant difference in serum immune enzyme activities among all the groups. The inconsistent research results may be attributed to the distinct experimental subjects and feed compositions. *E. sinensis* is omnivorous, when the FM in the diet substituted by DYM in an appropriate amount does not adversely affect the crabs. When the crabs are healthy, the immune-stimulating effect of chitin becomes less pronounced. However, when FM is completely replaced with DYM, a significant decline in the crabs' serum immune enzyme activity was observed, potentially due to excessive dietary chitin causing intestinal damage. The analysis of intestinal tissue structure and gut microbiota revealed that the DYM100 group exhibited a significant reduction in both the number and height of intestinal folds compared to the other groups. Additionally, there was a notable increasing gut microbiota abundance in the harmful bacterium *Shigella*. Thus, it is evident that the substitution of FM with DYM lead to a decline in nonspecific immune responses, potentially attributed to the detrimental impact of high chitin levels on the intestine.

GPT and GOT are two key enzymes in amino acid metabolism and vital indicators of normal liver function. These two enzymes are mainly present in the hepatopancreas, when liver function is impaired and the liver cell membrane is damaged, they are only released into the hemolymph [2]. In this study, the activity of serum GPT and GOT was unaffected by the content of DYM, and similar findings were reported in sea trout (*Salmo trutta m*. trutta, L.) [70] and mandarin fish [68]. Hence one can see that, the addition of dietary DYM did not affect the health of the crab hepatopancreas.

Tissue oxidative stress, which stimulates the production of reactive oxygen species (ROS), negatively affects the health and development of aquatic animals. Generally, antioxidant enzymes such as GSH-Px, CAT, SOD, are the main cellular protective mechanism against oxidative stress. In addition, T-AOC and MDA levels can also reflect the antioxidant capacity of aquatic animals. Previous study by Feng et al. [13] suggested that the serum SOD activities were significantly increased with the increasing proportion of YM in the diet of giant freshwater prawn, similar results can also be seen in the study of juvenile olive flounder (*Paralichthys olivaceus*) [71], mandarin fish [68], meagre fish (*Argyrosomus regius*) [72], and rainbow trout [73]. Conversely, a study by Jeong et al. [19] revealed that substituting 10%, 20%, 30%, and 40% of the FM in the diet of rainbow trout with full-fat YM, did not significantly affect the serum SOD and GSH-Px activities. In this experiment, when 25%, 50%, and 75% of dietary FM was replaced by DYM, there was no significant effect on the antioxidant capacity of crab serum. When dietary FM was completely replaced by DYM, the crab serum GSH-Px, T-AOC, and T-SOD activities were significantly reduced and MDA content was significantly increased compared to the CON group. Similar phenomenon has been reported by studies on Nile tilapia demonstrating that, when the supplementation of DYM reached 45%, the expression of oxidative stress-related genes nrf2 and klf9 in liver were significantly increased, resulting in the accumulation of ROS in the liver and significantly reduced the antioxidant capacity [74]. There are significant differences in the antioxidant response of aquatic animals to the addition of YM in the diet, which may be attributed to several factors, such as experimental subjects, growth stages, basic formulas, dosage of YM, and different treatment processes. In the future, extensive research should be conducted on the effects of YM on the antioxidant capacity of aquatic animals and delve into the underlying reasons and mechanisms of action.

## 5. Conclusion

In this experiment, DYM effectively substituted for 75% FM in the diet containing 200 g/kg FM without adverse effect on *E. sinensis*. However, when the substitution reaches 100%, there were significant adverse effects on the growth performance, intestinal health, serum immune, and antioxidant indexes of juvenile *E. sinensis*. In conclusion, the substitution level of FM with DYM was suggested to be 75% (150 g/kg).

## Figures and Tables

**Table 1:** Composition and nutrient levels of the experimental diets (air dry basis, g/kg).

Ingredients (%)	CON	DYM25	DYM50	DYM75	DYM100
FM	200.0	150.0	100.0	50.0	0
DYM	0	54.2	108.4	162.6	216.8
Flour	200.0	192.0	184.0	186.0	178.0
Corn	100.0	100.0	100.0	100.0	100.0
Meat and bone meal	100.0	100.0	100.0	100.0	100.0
Soybean meal	150.0	150.0	150.0	150.0	150.0
Peanut meal	50.0	50.0	50.0	50.0	50.0
Cottonseed meal	60.0	60.0	60.0	60.0	60.0
Squid visceral meal	40.0	40.0	40.0	40.0	40.0
Brewers dried yeast	20.0	20.0	20.0	20.0	20.0
Fish oil	20.0	23.8	27.6	21.4	25.2
Soyabean lecithin	20.0	20.0	20.0	20.0	20.0
Carboxymethylcellulose sodium	20.0	20.0	20.0	20.0	20.0
Mixed premix ^*∗*^	20.0	20.0	20.0	20.0	20.0
Total	1000.0	1000.0	1000.0	1000.0	1000.0
Proximate analyses					
Crude protein	375.3	383.1	381.6	378.2	374.4
Crude fat	88.9	84.3	89.1	85.5	84.6
Ash	83.5	83.4	86.7	89.1	86.5
Moisture	75.6	74.4	79.1	73.2	74.2

*Note:* Annotation  ^*∗*^1 kg of premix contained: vitamin A 8 × 104 IU, vitamin D_3_ 3.5–4 × 104 IU, vitamin E 0.6 g, vitamin K_3_ 0.2 g, vitamin B_1_ 0.1 g, vitamin B_2_ 0.3 g, vitamin B_6_ 0.16 g, vitamin B_12_ 0.4 mg, nicotinamide 0.8 g, calcium D, pantothenate 0.5 g, folic acid 0.05 g, D-biotin 1.6 mg, potassium 8.5 g, magnesium 1.2 g, iron 0.1–14 g, zinc 1–2.4 g, cuprum 0.3–1 g, manganese 0.3–3 g, cobalt 22–40 mg, selenium 0.36–10 mg, iodine 6–20 mg, inositol 3 g, vit C 3 g, choline chloride 50 g, moisture ≤10%.

Abbreviations: DYM, defatted yellow mealworm meal; FM, fish meal.

**Table 2:** Amino acids composition of the experimental diets, FM, and DYM (g/kg, air dry basis).

Ingredients	CON	DYM25	DYM50	DYM75	DYM100	FM	DYM
EAA							
Threonine	10.5	10.4	10.3	10.3	10.2	24.6	22.7
Methionine	7.7	7.5	7.3	7.1	7	17.6	13.2
Valine	8.1	8.3	8.5	8.6	8.8	35.6	39.4
Isoleucine	5.7	5.1	4.7	4.1	3.8	38.9	26.3
Leucine	29.3	30.1	30.9	31.5	32.2	30.6	47.0
Phenylalanine	32.7	32.5	32.3	32.0	31.8	33.2	28.3
Histidine	10.4	10	9.5	9.0	8.3	19.8	7.2
Lysine	20.2	20.1	20.3	20.2	20.0	48.7	48.9
Arginine	20.4	20.5	20.5	20.8	20.3	39.5	38.9
Tryptophan	9.2	9	8.9	8.8	8.6	6.4	3.8
NEAA							
Aspartic acid	33.2	32.4	31.7	30.9	30.3	61.8	45.7
Serine	20.3	20.5	21.2	22	22.9	24.5	40.3
Glutamic acid	65.1	64.1	63.2	63.2	62.4	92.6	70.1
Glycine	19.7	19.8	19.8	19.9	20.1	40.8	43.2
Alanine	21.6	21.0	20.4	19.9	19.3	43.2	31.3
Cysteine	1.8	1.6	1.6	1.5	1.4	4.9	3.6
Proline	21.2	22.4	23.4	24.3	25.7	27.6	52.5
Tyrosine	19.6	19.7	19.9	20.1	20.3	18.8	22.4
TAA	356.7	355	354.4	354.2	353.4	609.1	584.8

Abbreviations: EAA, essential amino acids; NEAA, nonessential amino acid; TAA, total amino acids.

**Table 3:** Effects of different substitution levels of FM with DYM on the growth performance of *E. sinensis*.

Item	Groups					Polynomial contrasts	
	CON	DYM25	DYM50	DYM75	DYM100	Linear	Quadratic
IBW (g)	4.96 ± 0.24	4.83 ± 0.26	4.93 ± 0.23	4.93 ± 0.23	5.06 ± 0.37	ns	ns
FBW (g)	18.21 ± 0.57^a^	18.03 ± 0.36^a^	18.04 ± 0.36^a^	17.48 ± 0.60^ab^	17.25 ± 0.46^b^	^*∗*^	ns
WG (%)	267.4 ± 13.4^a^	274.3 ± 14.5^a^	266.7 ± 15.3^a^	255.3 ± 15.9 ^ab^	241.7 ± 16.5^b^	^*∗*^	ns
FCR	2.01 ± 0.15^bc^	1.89 ± 0.17^c^	1.99 ± 0.18^bc^	2.22 ± 0.17^b^	2.51 ± 0.06^a^	^*∗∗∗*^	^*∗*^
SR (%)	77.94 ± 7.40	72.06 ± 7.40	73.53 ± 7.59	72.06 ± 5.63	75.00 ± 6.44	ns	ns
PER	1.43 ± 0.12^a^	1.36 ± 0.05^ab^	1.30 ± 0.09^ab^	1.29 ± 0.16^ab^	1.17 ± 0.03^b^	^*∗∗*^	ns
HSI (%)	8.64 ± 1.83	8.39 ± 1.65	7.91 ± 1.96	8.17 ± 1.81	7.63 ± 1.40	ns	ns
MI (d)	25.50 ± 0.58	25.25 ± 0.50	26.00 ± 0.82	26.25 ± 0.50	26.25 ± 0.96	ns	ns

*Note:* The data are expressed as mean ± standard deviation (*n* = 4).  ^*∗*^*P* < 0.05;  ^*∗∗*^*P* < 0.01;  ^*∗∗∗*^*P* < 0.001. In the same row, values with different superscripts mean significant difference (*P* < 0.05).

Abbreviations: DYM, defatted yellow mealworm meal; FBW, finial body weight; FCR, feed conversion ratio; FM, fish meal; HIS, hepatopancreas index; IBW, initial body weight; MI, molting interval; ns, no significant; PER, protein efficiency ratio; SR, survival rate; WG, weight gain rate.

**Table 4:** Effects of different substitution levels of FM with DYM on intestinal tissue structure of *E. sinensis*.

Item	Groups					Polynomial contrasts	
	CON	DYM25	DYM50	DYM75	DYM100	Linear	Quadratic
Number of folds	14.25 ± 0.96^a^	13.75 ± 0.96^a^	14.00 ± 1.15^a^	11.75 ± 0.96^a^	10.50 ± 1.29^b^	^*∗∗*^	ns
Folds height (μm)	204.50 ± 22.29^a^	202.75 ± 24.17^a^	210.50 ± 26.34^a^	208.50 ± 26.20^a^	148.50 ± 18.34^b^	^*∗*^	^*∗*^
Folds width (μm)	134.50 ± 18.08	136.75 ± 16.56	139.00 ± 13.49	136.25 ± 21.00	131.75 ± 22.71	ns	ns
Peritrophic membrane thickness (μm)	10.50 ± 1.29	11.00 ± 0.82	10.50 ± 1.29	11.25 ± 0.96	10.25 ± 1.50	ns	ns

*Note:* The data are expressed as mean ± standard deviation (*n* = 4).  ^*∗*^*P* < 0.05;  ^*∗∗*^*P* < 0.01. In the same row, values with different superscripts mean significant difference (*P* < 0.05).

Abbreviations: DYM, defatted yellow mealworm meal; FM, fish meal; ns, no significant.

## Data Availability

The data will be made available upon request.
